# The 100-most Cited Articles About Craniectomy and Hemicraniectomy: A Bibliometric Analysis

**DOI:** 10.7759/cureus.5524

**Published:** 2019-08-29

**Authors:** Eric Whitney, Deependra Mahato, Tiffany Odell, Yasir R Khan, Javed Siddiqi

**Affiliations:** 1 Neurosurgery, Desert Regional Medical Center, Palm Springs, USA

**Keywords:** bibliometric analysis, brain trauma, middle cerebral artery infarct, trephination, decompressive hemicraniectomy, craniectomy

## Abstract

Craniectomy is a life-saving procedure used in the setting of traumatic brain injury^, ^stroke and increased intracranial pressure. The purpose of this study was to analyze and determine the most influential articles and authors in the field of craniectomy. Our study presents an analysis of the articles that include the word "craniectomy" or "hemicraniectomy" in the title and a detailed analysis of the top 100-cited articles in that selection. This search provided insight into how this procedure was initially documented and how it has been utilized over the years. We used the SCOPUS database to search “craniectomy OR hemicraniectomy” in the article title. We then sorted the top 100 most-cited articles. Bibliometric analysis was performed. An H-index was presented with each author. The citation count ranged from 71 to 5310. The most published author was Werner Hacke, a German researcher (n=6). The highest quantity of influential work was published in 2006 and 2007 (n=9/yr). The United States published the most articles (n=42). The Journal of Neurosurgery published 21 of the top 100 most-cited articles. The chronological timeline shows the evolution of decompression as it related to both stroke and trauma. It demonstrated that well-cited articles acted as turning points to direct further scientific endeavors while highlighting the hard work of certain authors. There is, to the best of our knowledge, a shortage of literature on a bibliometric analysis regarding the term craniectomy. Thus, the current bibliometric study was undertaken to highlight the work of authors who have advanced knowledge about this procedure. It provides an analysis of the top 100-cited articles with craniectomy in the title with dates ranging from 1892 to 2016. A review of its publication history shows how interventions in this field have advanced over the last several decades.

## Introduction and background

A bibliometric analysis is the statistical analysis of written publications. It seeks to quantify the academic output of people and institutions. In the strictest sense, it is an effort to present an assessment of the scientific literature of a given topic. Understanding the publication data of a topic lends insight into the current state of that field and identifies those authors who have made the greatest impact. 

Craniectomy and hemicraniectomy are decompressive procedures wherein a bone flap is elevated from the skull to relieve intracranial pressure which has been refractory to medical management. The origin of craniectomy can be traced to the Neolithic period (9600 BC to 2000 BC) with the practice of trephination and the placement of burr holes into the cranium for various reasons. In fact, trephination is the earliest surgical procedure for which we have archaeological evidence [1]. Throughout history, craniectomy and hemicraniectomy have been associated with significant morbidity and mortality. Craniectomy has remained one of the most common neurosurgical procedures performed in traumatic brain injury in modern medicine. Nevertheless, the sequelae of increased intracranial pressure including stroke, herniation, and death remain. This procedure has been the target of several large-scale multicenter and single-center trials leading to its development and expansion with regards to determining its rates of morbidity and mortality. These trials include DECRA, DECIMAL, DESTINY I & II, HAMLET, RESCUEICP and more recently RESCUE-ASDH.

## Review

Methods

Elsevier’s Scopus, a comprehensive abstract and citation database for peer-reviewed literature, was primarily utilized for the collection of information about the publications under the search term "craniectomy" in May 2019. The search was conducted using the terms “craniectomy OR hemicraniectomy” in the article title. This resulted in 1,646 documents. These results were filtered for the top 100 most-cited articles. Using the Scopus analytics tool, we were able to view documents by year, documents per year by source, documents by author, documents by affiliation, documents by country, and documents by funding sponsor. The H index has been included for each top author. Originally proposed by Hirsch, it is a research output measure that represents original research and other publications designed to indicate impact [2]. For example, an H index value of 22 means that 22 articles have been cited at least 22 times. The 20 most-cited articles were selected for an in-depth review.

Results

**Figure 1:**
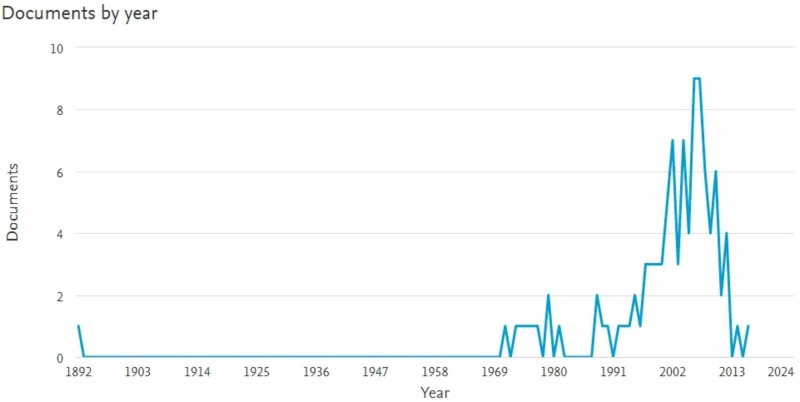
Graphs of the documents by year displaying a large growth in the 1990s and early 2000s

The documents by year analysis, from 1892 to 2016 (Figure 1), revealed a paucity of well-cited literature in the early 1900s. In 1892, a document titled, "Pioneer craniectomy for relief of mental instability due to premature sutural closure and microcephalus" was published and has garnered 132 citations to date [3]. In the 100 most-cited articles, there is a large time gap. The 1970s saw eight well-cited documents and outshined the 1980s, which only produced four of the top-cited articles. The 1990s saw a resurgence in publications (n=16). This trend continued into the 21st century as most of the top-cited articles were published between 2000 and 2009 (n=57). The past nine years have only seen 14 of the most-cited articles published with a dramatic taper from six in 2010 to one in 2016.

**Figure 2:**
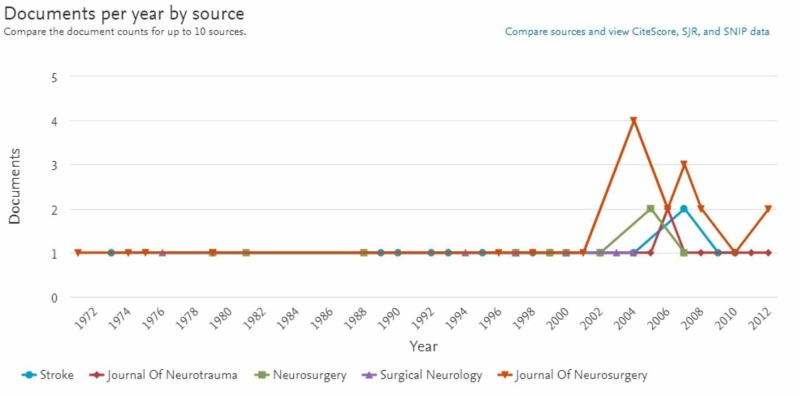
The five journals that have published the majority of the top-cited articles

The documents by source (Figure 2) analysis starts in 1972. Previous dates did not include the top sources. The Journal of Neurosurgery published 21 articles. The Stroke published 14; Neurosurgery following with 10. The Journal of Neurotrauma published nine and Surgical Neurology published six. These top three journals are responsible for 45% of the top-cited publications on this topic.

**Figure 3:**
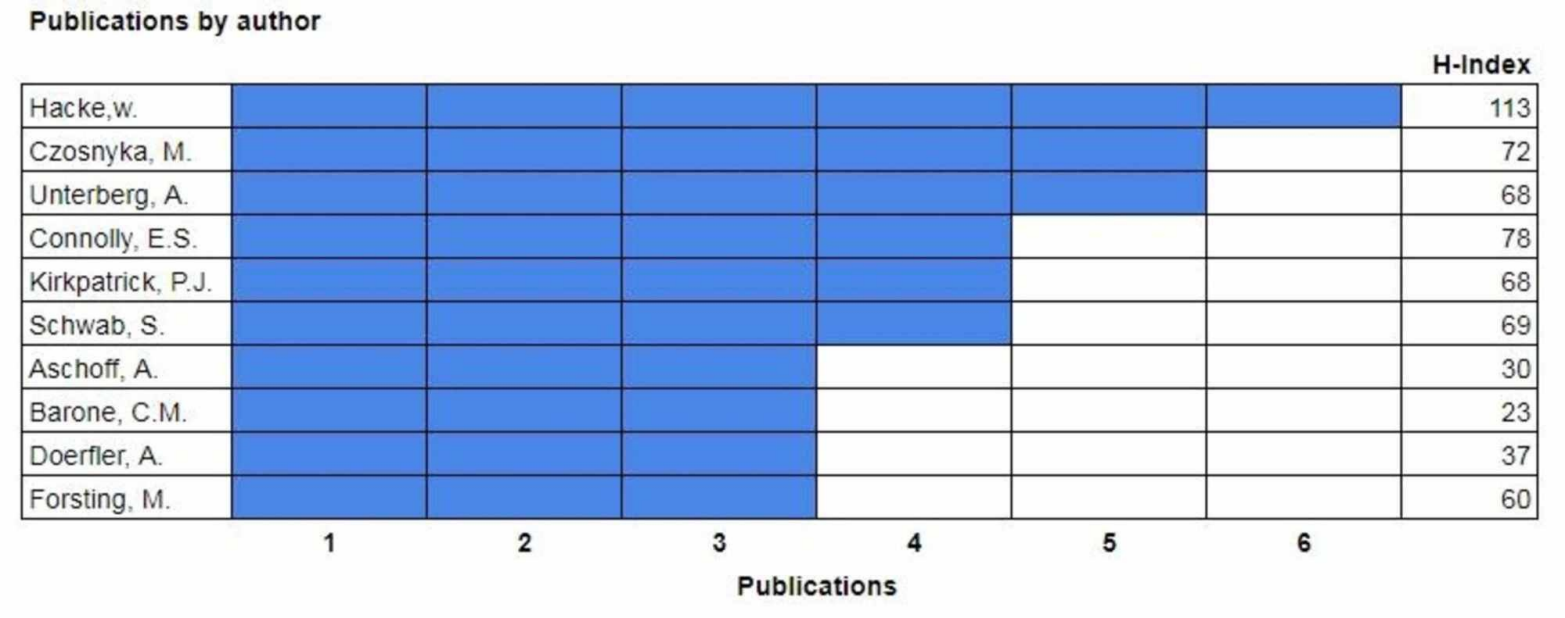
Authors responsible for the influencing the field

The documents by author category compare the publication count within the top 100 most-cited articles of specific authors and their H-index (Figure 3). 

The top-ranked author was Werner Hacke who published six of the most-cited articles. His most-cited article, "Early hemicraniectomy in patients with complete middle cerebral artery infarction" was published in 1998 and cited 552 times [4]. Two authors tied with five publications each: Czosnyka and Unterberg. Czosnyka’s paper, "Trial of decompressive craniectomy for traumatic intracranial hypertension", was published in 2016 and has been cited 237 times [5]. Unterberg’s article, "Hemicraniectomy in older patients with extensive middle-cerebral-artery stroke" was published in 2014 and has been cited 244 times [6]. Connolly, Kirkpatrick, and Schwab have each contributed four articles. Aschoff, Barone, Doerfler, Forsting, Hutchinson, Jimenez each contributed three articles.

The top authors were found to work in teams. The top-ranked author, Hacke, worked with the other well-ranked authors, namely, Doerfler, Forsting, Unterberg, and Schwab. Their work focused on craniectomy in the setting of stroke and encephalitis [4, 6-10]. Czosnyka worked with Hutchinson and Kickpatrick. Their work focused on decompressive craniectomy in traumatic brain injury and intracranial hypertension [5, 11-14]. Unterberg worked with Czosnyka, Hutchinson, and Kirkpatrick. Their publications from 2001-2016 focused on hemicraniectomy while considering intracranial hypertension, stroke, trauma, and functional outcomes [5-6, 11, 15-16]. Connolly was not published with the other top 10 authors (2002-2007). His worked highlighted hemicraniectomy in the setting of subarachnoid hemorrhage and infarctions [17-20]. Kirkpatrick worked with Czosnyka and Hutchinson (previously mentioned above) on publications from 2001 to 2016 and was affiliated with Addenbrooke Hospital and the University of Cambridge [5, 11, 13-14]. Schwab worked with Hacke and Aschoff producing works from 1997-2002 out of the University of Heidelberg [4, 10, 21-22]. Barone worked with Jimenez. His works, 1998-2004, focused on craniosynostosis and were affiliated with the University of Missouri-Columbia [23-25]. Doerfler worked with Forsting and produced top works from 1996-2002 in relationship to decompression in MCA stroke from the University of Heidelberg [7, 26-27].

**Figure 4:**
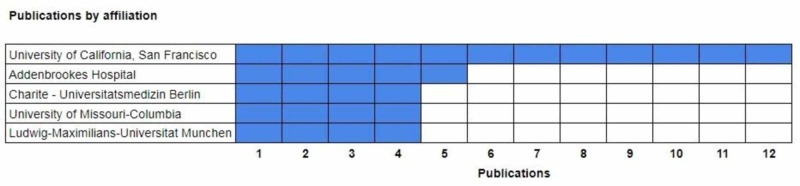
Listing of top institutions

The publications by affiliation category shows which institutions have produced the research (Figure 4). The University of California, San Francisco and its school of medicine produced 12 publications. The University of Heidelberg produced nine works [4-8, 10, 21-22, 28]. Addenbrooke’s Hospital produced five works [5, 11-14]. Charite Universitatsmedizin Berlin had four works. The University of Missouri-Columbia and Ludwig-Maximilians Universitat Munchen had three publications apiece [6, 15-16, 29].

**Figure 5:**
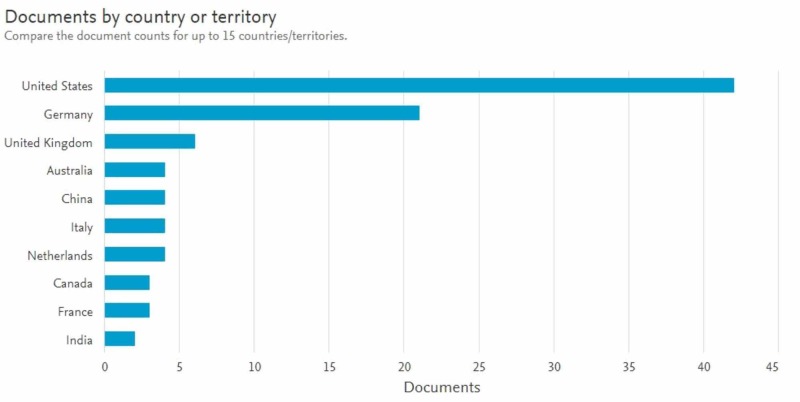
Graph showing the countries which produced the top articles

The United States contributed 42% of the literature on the topic, followed by Germany at 21%, and then United Kingdom at 8% (Figure 5).

The documents according to funding sponsors had three top contributions. Each entity had one publication. The work sponsored by Capital Foundation of Medical Development, a Chinese company, was cited 72 times [30]. The work by Deutsche Forschungsgemeinschaft, who funds medical research in Germany, was cited 245 times [6]. Medical Research Council, who coordinates medical research in the UK, had their article cited 237 times [5].

Discussion

**Figure 6:**
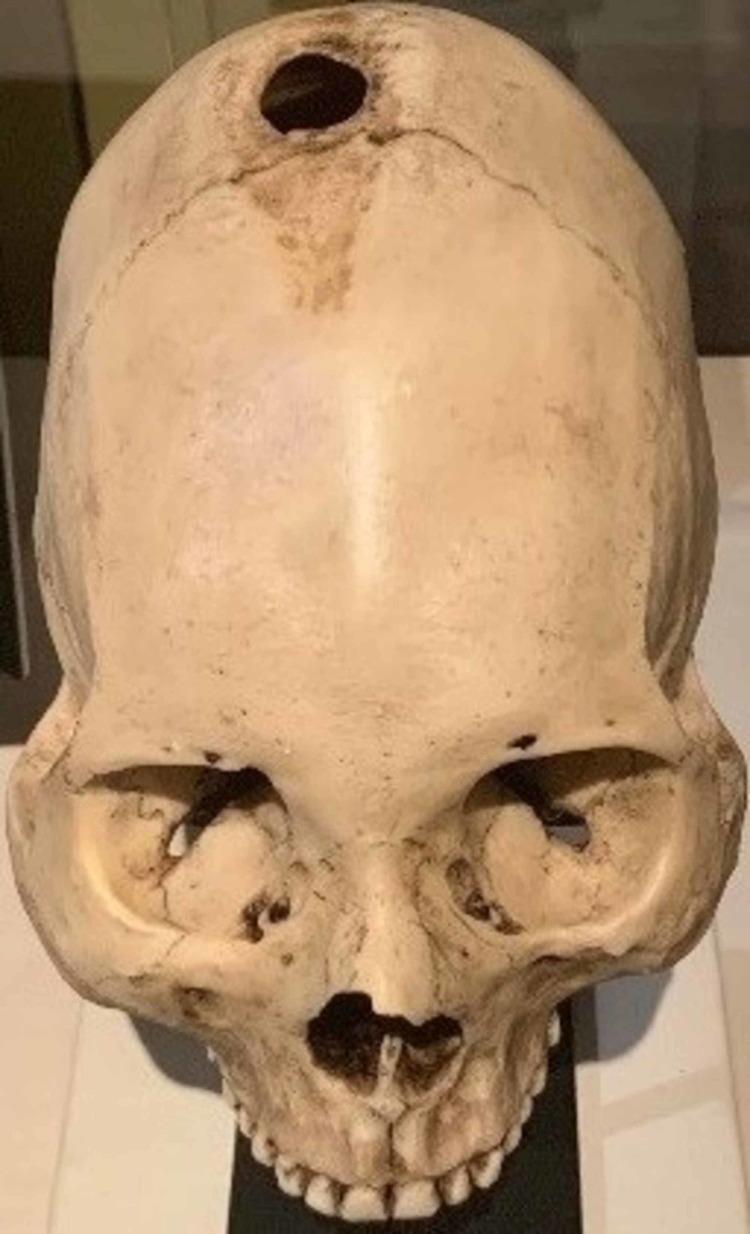
Photograph taken at the Houston Museum of Natural Science in June 2019 showing an early form of trephination Photo credit: Eric Whitney

The craniectomy procedure is a foundation of neurosurgical practice. Archaeological evidence of skulls from the Neolithic period demonstrating trephination, the ancient technique of burr holing (see Figure 6), make craniectomy the earliest surgical procedure recorded in history [1]. As surgical tools improved, the procedure also morphed from simple trephination into what is now known as craniectomy/craniotomy. In 1518, Berengario da Capri published a manuscript that describes both the indications and technique for craniotomy [31].

In 1892, Lane published a case series entitled "Pioneer craniectomy for relief of mental imbecility due to premature sutural closure and microcephaleus". In his brief article, he explains the circumstances under which he tried to “unlock the brain” of a 9-month-old. This child died 14 hours after the procedure due to anesthetic complications. A second patient from another case detailed in the same report was thriving at the time of his writing in 1892 [3]. There is a time gap from 1892 to 1971 where articles on these subjects did not make the list of top 100 most cited. After 1971, there was a noted emphasis on the application and development of craniectomy and are therefore highlighted in chronological order up until 1998, after which the publication load increased and the topic began to answer more specific questions.

In 1971, Ransohoff published his pioneering work on the use of hemicraniectomy in the management of acute subdural hematoma (n=35) in the Journal of Neurosurgery. He reported a 40% survival and 28% return to normal function. His results were outstanding for the time [32]. Subsequent utilization of the intervention failed to reproduce equivalent results and, in 1976, Cooper published (in Surgical Neurology) that in the five years proceeding Ransohoff’s paper only 50 patients were reported to be treated with decompressive craniotomy. Cooper reports a dismal 10% percent survival and 4% to function. He called for a move to restrict the utilization of the procedure [33]. Five years later (1981) in Neurosurgery Journal, Rengachary published a report of three patients treated with hemicraniectomy for “massive multilobar infarction […] who survived, although severe fixed neurological deficit persisted in two" [34]. In 1988, Kondziolka, citing Rengachary’s 1981 publication, reported on five patients who were permitted to progress to “uncal herniation and pending death” prior to surgery, achieved “good results” status post “decompressive craniectomy in the treatment of lesions causing increased intracranial pressure and brain edema” [35].

Delashaw argued in his 1990 Stroke publication based on a sample of nine that “hemicraniectomy can be an effective life-saving procedure for malignant cerebral edema after large hemispheric infarction” [36]. The year 1992 saw the advent of the first well-cited article on successful suboccipital craniectomy by Chen in Stroke [37]. In 1994 Fisher, in Surgical Neurology, published a case report discussing craniectomy in a comatose woman who suffered from a SAH [38]. By the mid-1990s, Hacke, Doerfler, Schwab, and Forsting were publishing high-ranking articles laying the groundwork for the trials of the 2000s (Table 1) [7-8, 10]. In 1995, Forsting published "Decompressive craniectomy for cerebral infarction: An experimental study in rats", which showed that decompression reduced mortality in rats and called upon neurosurgical intervention for this population. In 1997, Polin showed that “decompressive bifrontal craniectomy provides a statistical advantage over medical treatment of intractable posttraumatic cerebral hypertension and should be considered in the management of malignant post-traumatic cerebral swelling. If the operation can be accomplished before the ICP value exceeds 40 torr for a sustained period and within 48 hours of the time of injury, the potential to influence outcome is greatest [39]". 

**Table 1:** This lists the clinical trials related to decompressive craniectomy DECIMAL: decompressive craniectomy in malignant middle cerebral artery infarction; DESTINY: decompressive surgery for the treatment of malignant infarction of the middle cerebral artery; HAMLET: hemicraniectomy after middle cerebral artery infarction with life-threatening edema trial; DECRA: decompressive craniectomy trial; RESCUEicp: Randomized evaluation of surgery with craniectomy for uncontrollable elevation of intracranial pressure; RESCUE-ASDH: Randomized evaluation of surgery with craniectomy for patients undergoing evacuation of acute subdural hematoma

Trial Name	Citations
DECIMAL (2007): decompressive craniectomy in malignant middle cerebral artery infarction trial. They concluded in a population age 18-55 and N=38 an increase in moderate disability and a decrease in mortality when compared to medical therapy.	434
DESTINY (2007): decompressive surgery for the treatment of malignant infarction of the middle cerebral artery. It showed that there was a significant reduction in 30-day mortality for decompression as opposed to medical management.	484
HAMLET (2009): hemicraniectomy after middle cerebral artery infarction with life-threatening edema trial. They introduced a four-day window for surgical intervention (N=64). They concluded, "Surgical decompression reduces case fatality and poor outcome in patients with space-occupying infarctions who are treated within 48 hours of stroke onset. There is no evidence that this operation improves functional outcome when it is delayed for up to 96 h after stroke onset".	464
DECRA (2011): bifrontal temporoparietal decompressive craniectomy in diffuse traumatic brain injury for patients with refractory elevation in ICP was associated with decreased ICP but a higher incidence of overall morbidity.	733
DESTINY II (2014): Hemicraniectomy in older patients with extensive middle-cerebral-artery stroke.	247
RESCUEicp (2016): The trial of randomized evaluation of surgery with craniectomy for uncontrollable elevation of intracranial pressure after initial medical management was associated with lower mortality than medical management alone. Return of function with a need for higher level of dependent care was worse in the surgical group.	234
RESCUE-ASDH (2019) - Randomized evaluation of surgery with craniectomy for patients undergoing evacuation of acute subdural hematoma trial. Recently closed enrolment on May 1, 2019 after reaching the goal of 463 randomized patients	-

in 1998, Jimenez published, in the Journal of Neurosurgery, the article "Endoscopic craniectomy for early surgical correction of sagittal craniosynostosis". His four-case study report highlighted a decrease in blood loss, operative time, and cost with excellent surgical results [24]. That same year Schwab published "Early hemicraniectomy in patients with complete middle cerebral artery infarction". They stated that not only did craniectomy improve outcomes but also that early craniectomy (first 24 hours after onset of stroke) further improved outcomes [4]. In 2001, Holkamp published "Hemicraniectomy in elderly patients with space-occupying media infarction: Improved survival but poor functional outcome". They concluded that a large trial was needed to prove benefit [15].

Over the next 20 years, two major themes dominated. The first was decompression in the setting of infarction, and the second was decompression in the setting of trauma and increased intracranial pressure. Large clinical trials were undertaken to review the effectiveness of craniectomy (Table 1). Correlating to the published results of these trials is a large increase in year-by-year publications with the term in the title going from 42 papers in 2006 to 120 papers in 2016. Finally, in 2014 Juttler published DESTINY II, "Hemicraniectomy in older patient with extensive MCA stroke, to address the age limitations of previous trials". They concluded that hemicraniectomy improved the survival rates without severe disability in those 61 and older [6].

Limitations

All the top-100 articles with the term in the title were analyzed. However, it is difficult to ascertain which articles may have impacted the field but were simply not analyzed due to both search term limitations and the terms themselves. The ranking is subject to change as new publications are made available. 

## Conclusions

In this bibliometric analysis, we identified the top 100 most-cited articles with the term "craniectomy OR hemicraniectomy" in the title. The data presented shows the top contributors to the field and a chronological timeline in the evolution of these interventions by focusing on the articles that paved the way for clinical trials and more focused research.

## Appendices

These articles (Table 2) represent turning points and question generators that have helped shape the direction and discussion of the intervention.

**Table 2:** Top 20 most-cited articles

	Article Title	Cited by
1	Longa EZ, Weinstein PR, Carlson S, Cummins R. Reversible middle cerebral artery occlusion without craniectomy in rats. Stroke. 1989, 20:84-91. 10.1161/01.str.20.1.84	5310
2	Cooper DJ, Rosenfeld JV, Murray L, et al.: Decompressive craniectomy in diffuse traumatic brain injury. N Engl J Med. 2011, 364:1493-1502. 10.1056/NEJMoa1102077	733
3	Schwab S, Steiner T, Aschoff A, Schwarz S, Steiner HH, Jansen O, Hacke W: Early hemicraniectomy in patients With complete middle cerebral artery infarction. Stroke. 1998, 29:1888-1893. 10.1161/01.str.29.9.1888	552
4	Jüttler E, Schwab S, Schmiedek P, Unterberg A, Hennerici M, Woitzik J, Witte S, Jenetzky E, Hacke W: Decompressive surgery for the treatment of malignant infarction of the middle cerebral artery (DESTINY). Stroke. 2007, 38:2518-2525. 10.1161/strokeaha.107.485649	484
5	Hofmeijer J, Kappelle LJ, Algra A, Amelink GJ, van Gijn J, van der Worp HB: Surgical decompression for space-occupying cerebral infarction (the Hemicraniectomy After Middle Cerebral Artery infarction with Life-threatening Edema Trial [HAMLET]): a multicentre, open, randomised trial. Lancet Neurol. 2009, 8:326-333. 10.1016/s1474-4422(09)70047-x	464
6	Vahedi K, Vicaut E, Mateo J, et al.: Sequential-design, multicenter, randomized, controlled trial of early decompressive craniectomy in malignant middle cerebral artery infarction (DECIMAL Trial). Stroke. 2007, 38:2506-2517. 10.1161/strokeaha.107.485235	434
7	Polin RS, Shaffrey ME, Bogaev CA, Tisdale N, Germanson T, Bocchicchio B, Jane JA: Decompressive bifrontal craniectomy in the treatment of severe refractory posttraumatic cerebral edema. Neurosurgery. 1997, 41:84-94. 10.1097/00006123-199707000-00018	410
8	Aarabi B, Hesdorffer DC, Ahn ES, Aresco C, Scalea TM, Eisenberg HM: Outcome following decompressive craniectomy for malignant swelling due to severe head injury. J Neurosurg. 2006, 104:469-479. 10.3171/jns.2006.104.4.469	389
9	Taylor A, Butt W, Rosenfeld J, Shann F, Ditchfield M, Lewis E, Klug G, Wallace D, Henning R, Tibballs J: A randomized trial of very early decompressive craniectomy in children with traumatic brain injury and sustained intracranial hypertension. Childs Nerv Syst. 2001, 17:154-162. 10.1007/s003810000410	348
10	Sahuquillo J: Decompressive craniectomy for the treatment of refractory high intracranial pressure in traumatic brain injury. Cochrane Database Syst Rev. 2006. 10.1002/14651858.CD003983.pub2	265
11	Gupta R, Connolly ES, Mayer S, Elkind MSV: Hemicraniectomy for massive middle cerebral artery territory Infarction. Stroke. 2004, 35:539-543. 10.1161/01.str.0000109772.64650.18	253
12	Jüttler E, Unterberg A, Woitzik J, et al.: Hemicraniectomy in older patients with extensive middle-cerebral-artery Stroke. N Engl J Med. 2014, 370:1091-1100. 10.1056/NEJMoa1311367	244
13	Münch E, Horn P, Schürer L, Piepgras A, Paul T, Schmiedek P: Management of severe traumatic brain injury by decompressive craniectomy. Neurosurgery. 2000, 47:315-323. 10.1097/00006123-200008000-00009	241
14	Hutchinson PJ, Kolias AG, Timofeev IS, et al.: Trial of decompressive craniectomy for traumatic intracranial hypertension. N Engl J Med. 2016, 375:1119-1130. 10.1056/NEJMoa1605215	234
15	Delashaw JB, Broaddus WC, Kassell NF, et al.: Treatment of right hemispheric cerebral infarction by hemicraniectomy. Stroke. 1990, 21:874-881. 10.1161/01.str.21.6.874	203
16	Hutchinson PJ, Corteen E, Czosnyka M, et al.: Decompressive craniectomy in traumatic brain injury: the randomized multicenter RESCUEicp study (www.RESCUEicp.com). Brain Edema XIII. Acta Neurochirurgica Supplementum. Hoff JT, Keep RF, Xi G, Hua Y (ed): Springer, Vienna; 2006. 96:17-20. 10.1007/3-211-30714-1_4	197
17	Stiver SI: Complications of decompressive craniectomy for traumatic brain injury. Neurosurg Focus. 2009, 26. 10.3171/2009.4.focus0965	190
18	Gooch MR, Gin GE, Kenning TJ, German JW: Complications of cranioplasty following decompressive craniectomy: analysis of 62 cases. Neurosurg Focus. 2009, 26. 10.3171/2009.3.focus0962	187
19	Albanèse J, Leone M, Alliez J-R, Kaya J-M, Antonini F, Alliez B, Martin C: Decompressive craniectomy for severe traumatic brain injury: Evaluation of the effects at one year*. Crit Care Med. 2003, 31:2535-2538. 10.1097/01.ccm.0000089927.67396.f3	187
20	Yang XF, Wen L, Shen F, Li G, Lou R, Liu WG, Zhan RY: Surgical complications secondary to decompressive craniectomy in patients with a head injury: a series of 108 consecutive cases. Acta Neurochir (Wien). 2008, 150:1241-1248. 10.1007/s00701-008-0145-9	179
